# Characterization of the enteric virome of clinically healthy pigs around weaning on commercial farms in the Netherlands using next generation sequencing and qPCR

**DOI:** 10.1186/s40813-025-00446-5

**Published:** 2025-07-24

**Authors:** M.A.R. Schyns, R. van den Braak, J. Peijnenborg, S. Coppens, M. Deijs, M. G.J.M. Burggraaff, W. I. Kuller, S. Theuns, L. van der Hoek, A. de Groof

**Affiliations:** 1 Business Unit Intensive Livestock, MSD Animal Health Benelux, Wim de Körverstraat 35, Boxmeer, 5830 AA The Netherlands; 2 Department Discovery & Technology, MSD Animal Health, Wim de Körverstraat 35, Boxmeer, 5830 AA The Netherlands; 3 PathoSense BV, Pastoriestraat 10, Lier, 2500 Belgium; 4 Laboratory of Experimental Virology, Department of Medical Microbiology and Infection Prevention, Amsterdam UMC, University of Amsterdam, Meibergdreef 9, Amsterdam, 1105 AZ The Netherlands; 5 Amsterdam Institute for Infection and Immunity, Postbus, Amsterdam, 22660, 1100 DD The Netherlands; 6 University Farm Animal Practice, Reijerscopse Overgang 1, Harmelen, 3481 LZ The Netherlands

**Keywords:** NGS, Virome dynamics, Enteric viruses, Rotavirus, Astrovirus, Kobuvirus, Rectal swabs

## Abstract

**Background:**

Enteric virus infections around time of weaning have always been related to pig diseases such as postweaning diarrhea. Little, however, is known about the virus infection pattern (species, timing and viral load) in clinically healthy pigs. Virus infections may help to train and shape the immune system and presumably only lead to clinical disease when uncontrolled. Next Generation Sequencing (NGS) is a relatively new technique that can uncover the composition of the enteric virome. This study describes the dynamics of the enteric virome in clinically healthy pigs using NGS and qPCR until 10 weeks of age.

**Methods:**

Seven farms were selected based on the following criteria: diarrhea after weaning was visible in less than 5% of the pens, piglets reached 25 kg of body weight before 10 weeks of age and no antimicrobial batch treatment had been used on the farm for the last six months. Rectal swabs were taken in five different age groups: 2, 3.5, 5, 7 and 10 weeks of age, 10 piglets per age group, in a cross-sectional setup. Two NGS platforms were used to detect enteric viruses. Eleven virus-specific qPCRs were used to corroborate the results of the NGS analyses.

**Results:**

Rotavirus A, Porcine Kobuvirus, Enterovirus G and Porcine Astrovirus 3 and 4 were first detected at two weeks of age, followed by detection of Porcine Astrovirus 5 at 3.5 weeks of age, just before weaning. One week after weaning, at 5 weeks of age, Porcine Astrovirus 3 was undetectable, but now Porcine Astrovirus 1 and 2 had successively made their entry. Although Rotavirus B & C, Porcine Sapelovirus and Porcine Sapovirus were already detected just before weaning, the amount of virus peaked one week after weaning. Rotavirus H was first detected one week after weaning and peaked at 7 weeks of age. Many viruses were cleared by the age of 10 weeks.

**Conclusions:**

The timing and magnitude of subclinical enteric virus infections across farms were remarkably similar. Our study offers insight into the dynamics of enteric virome development in healthy pigs and provides essential context to NGS-based diagnostics.

**Supplementary Information:**

The online version contains supplementary material available at 10.1186/s40813-025-00446-5.

## Background

The postweaning period is one of the most critical stages in the life of pigs, with sudden social, environmental, nutritional and immunological changes that significantly impact health, welfare and performance. Diseases affecting the intestinal tract are amongst the most important economic problems affecting pork production [1]. Bacteria, parasites or viruses can cause enteric disease as a single infectious agent, but multiple concurrent and sequential infections may also occur, especially when the immune system is already heavily challenged. Enteric viral infections around time of weaning have been linked to diseases such as postweaning diarrhea [2–4]. However, the same viruses are also detected in healthy pigs, complicating the interpretation of causation. Current diagnostics mostly focus on the detection of single viruses or bacteria during clinical disease. They tend to ignore the diversity of infectious agents present in clinically healthy animals that grow well, as well as the infection dynamics of these viruses and bacteria. It is now accepted that– besides the well-known microbiome–, also a complex enteric virome develops in early life [5]. Despite advances in technology, there is still limited knowledge on the exact timing and magnitude of enteric viral infections that occur in clinically healthy pigs in the first 10 weeks of life.

Novel technologies for detection of viruses, especially Next Generation Sequencing (NGS), provide a wealth of information on the simultaneous presence of different viruses in piglets and pigs. Unfortunately, NGS has also made the interpretation of a specific pathogen’s presence in the context of disease more difficult. The detection of a particular virus in a rectal swab from a diseased pig does not immediately prove its relationship with disease, as it must be interpreted in the context of other pathogens present. Several NGS-based studies have been conducted to compare enteric viromes of healthy and diarrheic piglets to relate presence of specific viruses to diarrhea [2, 5]. Although candidates were identified, it proved necessary to analyze histopathological lesions that are characteristic of diarrhea to confirm the relation between the virus candidate and the clinical signs. Even with histological investigation, it is sometimes difficult to confirm this relationship with disease. For example Rotavirus A (RVA) can replicate and cause diarrhea without strong histopathological lesions due to enterotoxin (NSP4) production leading to malabsorption and hypersecretion [6]. Folgueiras et al. [7] specifically compared enteric viromes of wasting and non-wasting piglets at different time points of their life. While this study gave insight into the composition of the enteric virome of diseased pigs, it also revealed that viruses which have been directly related to enteric disease are readily detected in healthy pigs.

This study applied two NGS methods to characterize the enteric virome of farms with clinically healthy pigs in the Netherlands in the weeks before and after weaning. To verify the dynamics and changes in the enteric virome, multiple qPCRs were used to quantify the viral load in the samples and corroborate the NGS results. The results show the dynamics of ‘pathogenic’ viruses in clinically healthy herds and provide relevant context for interpretation of NGS and qPCR data obtained from pigs with clinical enteric disease.

## Methods

### Farm selection and study design

A total of seven farms were selected and labeled “well-performing” based on the following criteria:

Diarrhea after weaning was visible in less than 5% of the pens, piglets reach 25 kg of bodyweight before 10 weeks of age, and no antimicrobial batch treatment was used for the last six months to treat pigs after weaning. The selection of the farms was based on the history provided by the herd veterinarian. Clinical status and the use of antimicrobials was confirmed by a farm visit by the herd veterinarian at least every four weeks in a six-month period before sampling. The sampling was performed by a veterinarian who confirmed the clinical status during the collection of the samples.

In the first four farms, the sampling strategy entailed that 10 rectal swabs were taken from pigs a few days (3–5) before weaning (3.5 weeks of age), 10 rectal swabs from pigs one week after weaning (5 weeks of age), and 10 rectal swabs from pigs 3 weeks after weaning (7 weeks of age). For each age group, swabs were taken from 2 piglets in the same litter/pen, in total 5 separate litters/pens. A cross-sectional set-up was chosen in which the 30 samples were collected at the same time. Because of a 4-week batch system on one farm, one group was sampled two weeks after the first sampling.

After the NGS results (both VIDISCA and nanopore) of farms 1–4 had been analyzed, the study design was extended to include another three farms on which, besides the sampling described above, 10 rectal swabs of pigs were taken from piglets at 2 weeks of age (2 swabs per litter from 5 litters and 10 rectal swabs from pigs at 6 weeks after weaning (10 weeks of age, 2 swabs per pen, 5 pens in total). In total, 50 samples were collected on these farms. Also here, due to a 4-week batch system, the samples were collected in two sessions at one farm. An overview of the samples collected, and the analyses performed is displayed in Table 1. Note that only nanopore NGS analysis was performed on farms 5–7.

No vaccinations were given against the pathogens further investigated in this study by qPCR except for Rotavirus. In farms 1, 5 and 7, sows were vaccinated with a vaccine containing bovine Rotavirus at the end of gestation. No vaccination was given to the piglets against Rotavirus on any of the farms.

**Table 1:** Overview of samples collected from piglets and analyses performed

Analyses performed	Age				
	2 weeks	3.5 weeks	5 weeks	7 weeks	10 weeks
VIDISCA-NGS		Farms 1–4 (samples 1–8)	Farms 1–4 (samples 1–8)	Farms 1–4 (samples 1–8)	
Nanopore sequencing	Farms 5–7 (samples 1–10, pooled by 5)	Farms 1–7 (samples 1–10, pooled by 5)	Farms 1–7 (samples 1–10, pooled by 5)	Farms 1–7 (samples 1–10, pooled by 5)	Farms 5–7 (samples 1–10, pooled by 5)
qPCR	Farms 5–7 (samples 1–10)	Farms 1–7 (samples 1–10)	Farms 1–7 (samples 1–10)	Farms 1–7 (samples 1–10)	Farms 5–7 (samples 1–10)

### Sample collection

Rectal samples were collected using swabs (MWE, Corsham, UK) and immediately stored in 1 mL Sigma Virocult medium (MWE, Corsham, UK). Samples were transported to the laboratory at ambient temperature and stored at 2–8 °C until processed on the same day. In brief, samples were vortexed, transferred to 1.5 mL Eppendorf tubes, and centrifuged for 10 min at 10,000× g at 4 °C. Supernatants of the samples were subsequently stored at − 80 °C until further processing.

### VIDISCA-NGS

Rectal swab samples collected at farms 1–4 were analyzed using Virus Discovery cDNA-AFLP (VIDISCA) combined with NGS as described by de Vries et al. [8]. Of each sampling set per time point (n = 10 samples) the first 8 were chosen for VIDISCA-NGS. This selection (8 of 10) was due to space limitation per VIDISCA run. In short, 110 µL processed rectal swab suspension sample in Virocult (MWE, Corsham, UK) was centrifuged at 5000× g for 10 min and the supernatant was treated with TURBO™ DNase (Thermo Fisher Scientific, Waltham, MA, USA). Thereafter, nucleic acids were isolated by the Boom method [9], followed by reverse transcription with non-ribosomal random hexanucleotides [10] and second DNA strand synthesis with Klenow Fragment (3’5’ exo-) (New England Biolabs, Ipswich, MA, USA) and RNase H (Amersham Pharmacia Biotech, Amersham, UK) followed by a phenol chloroform extraction and ethanol precipitation. DNA was digested with MseI (TˆTAA; New England Biolabs, Ipswich, MA, USA) and ligated to adaptors incorporating a sample identifier sequence. DNA fragments with lengths above 200 bp were then purified by size selection with AMPure XP beads (Beckman Coulter, Brea, CA, USA). Next, a 28-cycle PCR with adaptor-annealing primers was performed, followed by size selection purification to continue only with fragments between 200 bp and 500 bp (AMPure XP beads). DNA concentration and fragment length of the libraries was assessed using Quant-it dsDNA HS Qubit kit (Thermo Fisher Scientific Waltham, MA, USA) and the Bioanalyzer (High Sensitivity Kit, Agilent Genomics, Santa Clara, CA, USA), respectively. Seventy sample libraries were pooled at an equimolar concentration [8, 11, 12] and sequencing was subsequently performed on an Ion Torrent PGM™ platform or the S5 platform (Thermo Fisher Scientific, Waltham, MA, USA) using the ION 316 Chip (PGM™ platform) or the ION 510 Chip (S5 platform) with 400 bp read length and 2 million sequences per run. To identify and classify viral reads and background, the obtained reads were translated into protein sequences and then aligned to the NCBI eukaryotic viral Identical Protein groups using UBLAST and analyzed with the VIDISCA bioinformatics workflow [13]. Classification of a viral read was performed by aligning the initial VIDISCA reads at the nucleotide level to a virus reference sequence database using CodonCode Aligner (version 6.0.2). Sequences with a viral reference hit above 80% were classified as said virus. The number of viral reads per sample was established as the sum of VIDISCA reads aligned to a virus reference sequence.

### NGS approach using nanopore sequencing

Ten samples of an individual sample point were pooled in groups of five and subsequently analyzed by PathoSense BV (Lier, Belgium) using nanopore sequencing (Oxford Nanopore Technologies). NGS analysis was performed on each pool as described previously by Vereecke et al. [14]. In general, the sample was purified & filtered using a centrifugation filter (Vivaclear Mini Clarifying Filter 0.8 μm, Sartorius) to discard host cells. An internal control virus was added to the samples as a measure of quality control and to facilitate semi quantification in the final data analysis. The samples were submitted to Benzonase nuclease treatment and nucleic acid extraction. Next, reverse transcription and limited DNA/RNA amplification were performed using an in-house developed workflow developed by PathoSense, as described previously [15–18]. Metagenomic sequencing was done on a GridION X5 (ONT) sequencing device using R10.4.1 flow cells (ONT) in combination with a Rapid Barcoding Kit (SQK-RBK114-24, ONT). Reads were base called using the “super accurate” base calling model in Guppy (v7.1.4; ONT). This workflow enabled the identification of both DNA/RNA viruses and bacteria in an at random manner via the taxonomical classification of the reads against a curated database. In the current study, the results analysis exclusively focused on the enteric virome. To exclude false positive and aspecific hits, host material was removed using the Sus scrofa reference genome (SusScr11) along with an additional validation against the complete NCBI nucleotide database. The results were reported semi quantitatively (not detected, very low, low, medium, high and very high) by normalizing each virus’s output against that of the previously added spike-in virus as described previously [15]. For calculation of a farm average the score was converted to a numeric scale where “not detected” was given a score 0 and a “very high” a score 5. Astroviruses were classified as pAstV1-5 based on the paper of Xiao et al. [19], which is different from the ICTV classification. If enough data of Rotavirus A (RVA) was present in samples from the nanopore sequencing, the RVA strains were further classified according to the whole genome-based classification method by Matthijnssens et al. [20] and Theuns et al. [21].

### Rotavirus A & C detection by multiplex RT-qPCR

For RNA extraction, 20µL of the processed rectal swab suspension sample in Virocult was used, which was diluted in 180µL PBS before extraction. Nucleic acids were extracted by the automated MagNA Pure 96 system (Roche Applied Science, Mannheim, Germany) using the protocol ‘Viral NA plasma external lysis SV3.1’. After extraction, the RNA was converted to cDNA using the QuantiTect Reverse Transcription Kit from Qiagen according to manufacturer protocol. The obtained cDNA was diluted 10 × (20µL cDNA and 180µL WFI (water for injection)). The RVA and Rotavirus C (RVC) RT-qPCR multiplex assay was performed as described by Marthaler et al. [22]. Primers and hydrolysis probes were designed on RVA and RVC VP6 sequences. The Advanced Universal Probes Supermix (Bio-Rad Laboratories, Hercules, CA, USA) was used for the RVA & RVC RT-qPCR assay as follows. Reactions were performed in a final volume of 25 µL. The mix used was: 12.5µL SsoAdvanced, 2µL WFI, 0.75µL RVA Fw (20µM), 0.75µL RVA Rv (20µM), 1µL RVA probe A1 (10µM), 0.5µL RVA probe A2 (10µM), 0.75µL RVC Fw (20µM), 0.75µL RVC Rv (20µM), 1µL RVC probe (10µM) and 5µL of the 10 x diluted cDNA. Primer and probe sequences are shown in Additional file 1: Table S1. Thermocycling was performed in the CFX96 Touch real-time PCR detection system (Bio-Rad Laboratories, Hercules, CA, USA) starting with the pre-denaturation 3 min at 95 °C, followed by 40 cycles of 15 s at 95 °C (denaturation) and 30 s at 60 °C (annealing and elongation). Results were analyzed with the CFX Maestro software (Bio-Rad Laboratories, Hercules, CA, USA). Lower limit of detection of the viral load in the samples was set at 10^4 copies/mL.

### Astrovirus detection by multiplex RT-qPCR

Two multiplex RT-qPCR assays, one for the detection of Porcine Astrovirus 1 (PAstV1) and 2 (PAstV2) and one for the detection of Porcine Astrovirus 3 (PastV3), 4 (PAstV4), and 5 (PAstV5), were adapted from Xiao et al. [19]. The SsoAdvanced Universal Probes Supermix (Bio-Rad Laboratories, Hercules, CA, USA) was used in the assays. The PAstV1-2 RT-qPCR reactions were performed in a final volume of 25 µL containing 12.5µL SsoAdvanced, 5.5µL WFI, 0.5 µL PAstV forward primer (10µM), 0.5 µL PAstV reverse primer (10µM), 0.5 µL PAstV1 probe (10µM), 0.5 µL PAstV2 probe (10µM) and 5 µL of the 10x diluted cDNA. Primer and probe sequences are shown in Additional file 1. Thermocycling was performed in the CFX96 Touch real-time PCR detection system (Bio-Rad Laboratories, Hercules, CA, USA) starting with the Pre-denaturation for 3 min at 95 °C, followed by and 40 cycles of 15 s at 95 °C (denaturation) and 30 s at 55 °C (annealing and elongation). Results were analyzed with the CFX Maestro software (Bio-Rad Laboratories, Hercules, CA, USA). The PAstV3-4-5 RT-qPCR reactions were performed in a final volume of 25 µL containing 12.5µL SsoAdvanced, 5µL WFI, 0.5 µL PAstV forward primer (10µM), 0.5 µL PAstV reverse primer (10µM), 0.5 µL PAstV3 probe (10µM), 0.5 µL PAstV4 probe (10µM), 0.5 µL PAstV5 probe (10µM), and 5 µL of the 10 x diluted cDNA. Primer and probe sequences are shown in Additional file 1: Table S1. Thermocycling was performed as described before for the PAstV1-2 RT-qPCR. Lower limit of detection of the viral load in the samples was set at 10^4 copies/mL.

### Porcine kobuvirus, Porcine sapelovirus, enterovirus G and Porcine Sapovirus detection by RT-qPCR

The Porcine Kobuvirus (PKoV) RT-qPCR was designed based primers/probe from Zhou et al. 2016 [22]. Porcine Sapelovirus (PSV), Enterovirus G (EV-G) and Porcine Sapovirus (PSaV) assays were designed specifically for the current study. Primers/probe sequences are shown in Additional file 1: Table S1. The location of PCR-primer annealing was chosen on conserved regions of the genomes, whereas the probe-sequences were designed after Sanger sequencing of PCR products obtained from farms 1–4. The PKoV, PSV, EV-G and PSaV assays were performed using the SsoAdvanced Universal Probes Supermix (Bio-Rad Laboratories, Hercules, CA, USA). The RT-qPCR reactions were performed in a final volume of 25 µL containing 12.5µL SsoAdvanced, 6.5µL WFI, 1 µL PrimeTime qPCR Assay (25x stock concentration, 5µM primer, 2.5µM probe) and 5 µL of the 10 x diluted cDNA. Thermocycling was performed in the CFX96 Touch real-time PCR detection system (Bio-Rad Laboratories, Hercules, CA, USA) starting with the Pre-denaturation for 3 min at 95 °C, followed by and 40 cycles of 15 s at 95 °C (denaturation) and 45 s at 60 °C (annealing and elongation). Results were analyzed with the CFX Maestro software (Bio-Rad Laboratories, Hercules, CA, USA). Lower limit of detection of the viral load in the samples was set at 10^4 copies/mL.

### Statistics analysis

Statistical analyses on viral read counts obtained by VIDISCA (Farms 1–4) were performed using GraphPad Prism 10.2.2. The cumulative read counts per farm were calculated and used to determine percentages of reads found per age group (3.5, 5 & 7 weeks of age). Percentages were calculated to correct for farm effects. Unpaired T-tests were used for the comparison between the age groups across farms. Statistical significance was set to *p* < 0.01 or lower.

## Results

### Enteric Virome analysis using VIDISCA-Next generation sequencing

To assess the composition of the enteric virome, we employed the VIDISCA-Ion Torrent next-generation sequencing (NGS) platform. This method is capable of detecting known viruses and viral sequences with low similarity to known strains. Although VIDISCA is a labor-intensive technique and not intrinsically quantitative due to an amplification step in sample processing, it allows for a comprehensive overview of the circulating viruses in herds around the time of weaning, including the potential identification of novel viral sequences. We chose to analyze a subset of individual samples (Farms 1–4) taken just before weaning (3.5 weeks of age), one week after weaning (5 weeks of age), and three weeks after weaning (7 weeks of age) using this NGS method. For each time point, the first eight samples from each age group were analyzed, a total of 96 samples (Table 1). The following viruses were identified: Rotavirus A (RVA), Rotavirus B (RVB), Rotavirus C (RVC), Rotavirus H (RVH), Porcine Astrovirus (PAstV), Porcine Kobuvirus (PKoV), Porcine Sapelovirus (PSV), Enterovirus G (EV-G), Porcine Sapovirus (PSaV), Porcine Bocavirus (PBoV), Porcine Torovirus (ToV), Picobirnavirus (PbiV), Porcine Teschovirus (PTV), and Posavirus (PoV). A summary of the characteristics of these viruses is presented in Table 2. Notably, no novel viruses were detected in any of the samples analyzed.

**Fig. 1:**
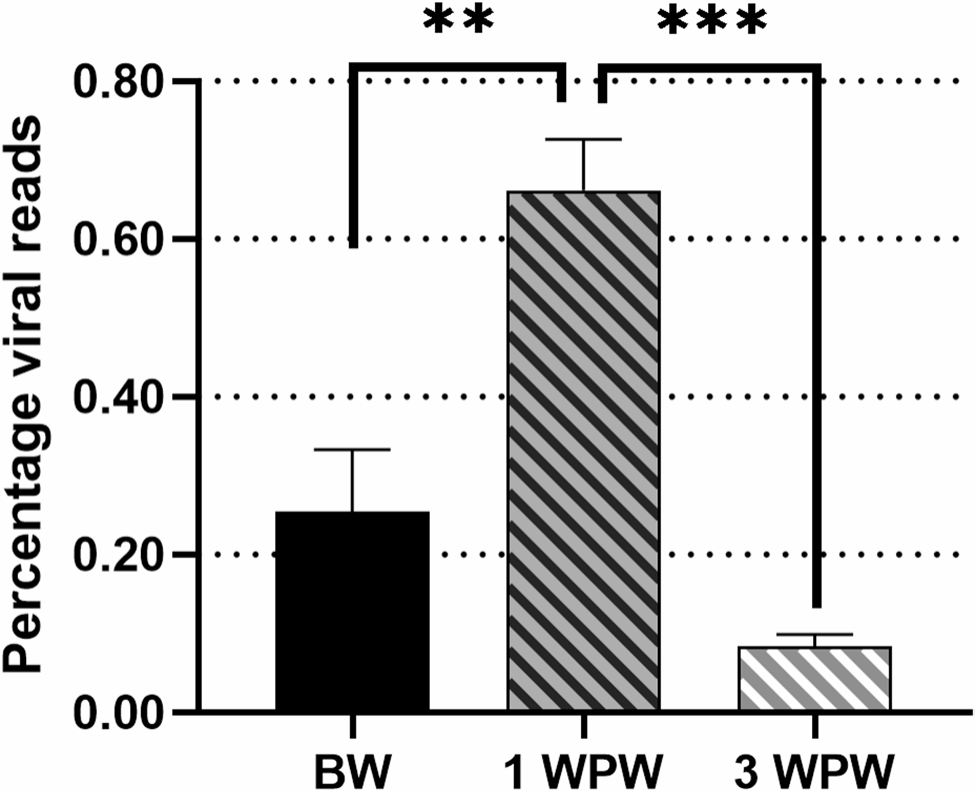
The percentage of viral reads detected in piglets by VIDISCA NGS per age group. BW = before weaning (3.5 weeks of age) 1 WPW = 1 week after weaning (5 weeks of age) 3 WPW = 3 weeks after weaning (7 weeks of age). Mean + SEM is shown. ** (*p* < 0.01). *** (*p* < 0.001), unpaired T-test

Calculated from the total number of viral reads, 25.5% of the reads were detected before weaning, and 66.1% one week after weaning (*p* < 0.01) (see Fig. 1). Three weeks post-weaning, the percentage read counts decreased to 8.4%, which was significantly lower compared to the one-week post-weaning group (*p* < 0.001).

**Table 2:** Characteristics of the porcine viruses detected by VIDISCA-NGS

Abbreviation	Common name	Order	Genus/species according ICTV	Enveloped virus	Viral genome structure	Genome size (kB)
RVA	Rotavirus A	Reovirales	Rotavirus alphagastroenteritidis	No	double-stranded segmented RNA	18–19
RVB	Rotavirus B	Reovirales	Rotavirus betagastroenteritidis	No	double-stranded segmented RNA	18–19
RVC	Rotavirus C	Reovirales	Rotavirus tritogastroenteritidis	No	double-stranded segmented RNA	18–19
RVH	Rotavirus H	Reovirales	Rotavirus aspergastroenteritidis	No	double-stranded segmented RNA	18–19
PAstV	Porcine Astrovirus	Stellavirales	Mamastrovirus	No	single-stranded positive-sense RNA	6.4–7.7
PKoV	Porcine Kobuvirus	Picornavirales	Kobuvirus cebes	No	single-stranded positive-sense RNA	8.2–8.3
PSV	Porcine Sapelovirus	Picornavirales	Sapelovirus anglia	No	single-stranded positive-sense RNA	7.5–8.3
EV-G	Enterovirus G	Picornavirales	Enterovirus geswini	No	single-stranded positive-sense RNA	7.5
PSaV	Porcine Sapovirus	Picornavirales	Sapovirus sapporoense	No	single-stranded positive-sense RNA	7–8
PBoV	Porcine Bocavirus	Piccovirales	Bocaparvovirus	No	single stranded (mostly) negative-sense DNA	4–6
ToV	Porcine Torovirus	Nidovirales	Torovirus suis	No	single-stranded positive-sense RNA	25–30
PbiV	Picobirnavirus	Durnavirales	Orthopicobirnavirus	No	double-stranded segmented RNA	3.7–4.5
PTV	Porcine Teschovirus	Picornavirales	Teschovirus asilesi	No	single-stranded positive-sense RNA	7.1
PoV	Posavirus	Picornavirales	Picornavirus	No	single-stranded positive-sense RNA	10

### Nanopore sequencing analysis of enteric Viromes on the complete sample set

We continued to analyze the complete sample set, now including Farms 5–7, using a nanopore sequencing platform from Oxford Nanopore Technologies, which is available to veterinarians for diagnostic purposes. As anticipated, the viral species identified included Rotavirus A (RVA), Rotavirus B (RVB), Rotavirus C (RVC), Rotavirus H (RVH), Porcine Astrovirus types 1–5 (PAstV1-5), Porcine Kobuvirus (PKoV), Porcine Sapelovirus (PSV), Enterovirus G (EV-G), Porcine Sapovirus (PSaV), Porcine Bocavirus (PBoV), Porcine Torovirus (ToV), Picobirnavirus (PbiV), Porcine Teschovirus (PTV), and Posavirus (PoV). These findings corroborated the enteric virome composition obtained through VIDISCA-NGS. In addition, we incidentally detected Porcine Parvovirus type 2 (PPV2) and type 4 (PPV4), as well as Porcine Adenovirus (PAV), although these were present at very low levels.

**Fig. 2:**
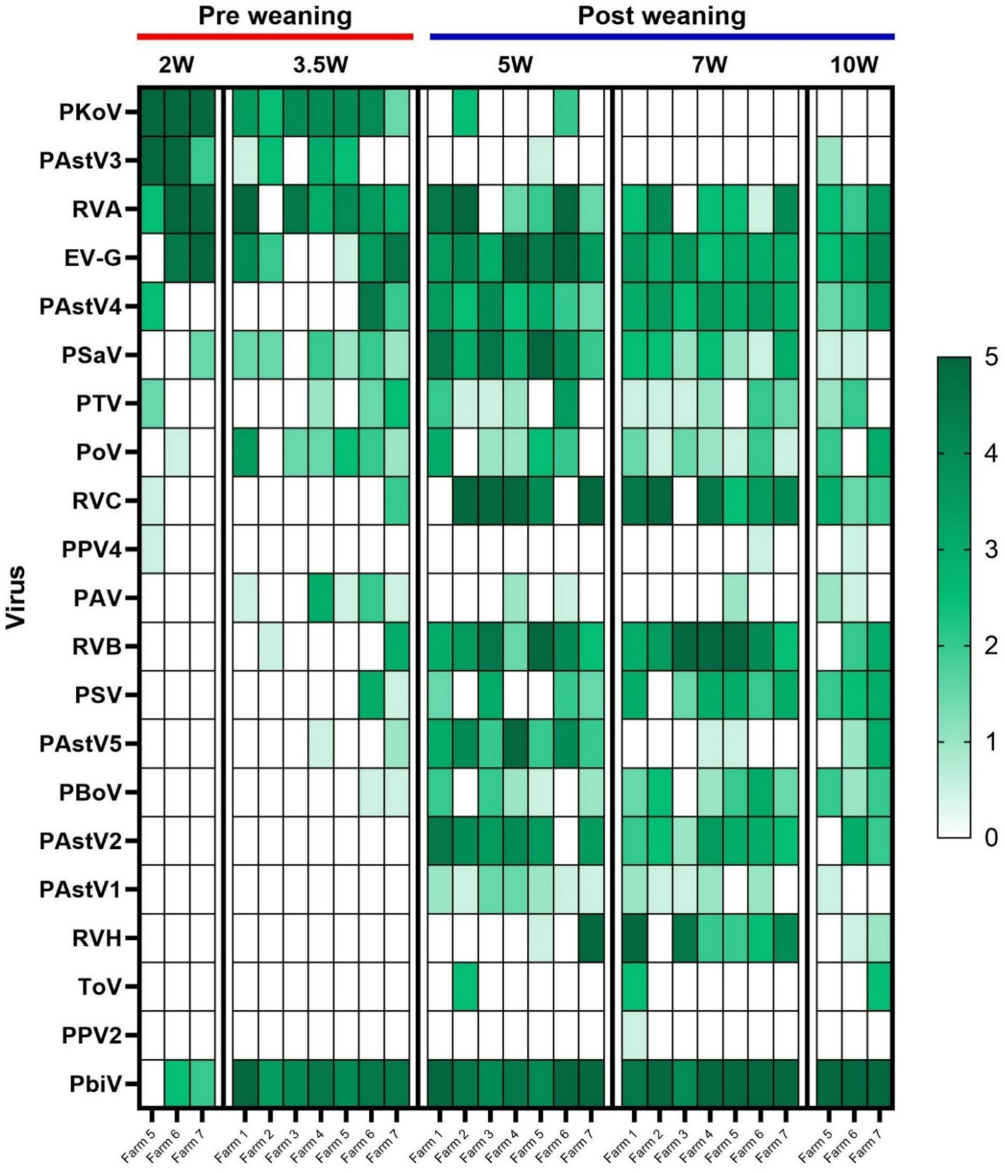
Average score of the semi-quantative nanopore sequencing results in piglets for each virus at different ages (cross-sectional sampling was performed). Not detected = 0, very low = 1, low = 2, medium = 3, high = 4 and very high = 5, based on normalizing the virus output to that of a spiked virus (details are explained in materials and method). Porcine Kobuvirus (PKoV), Porcine Astrovirus 3 (PAstV3), Rotavirus A (RVA), Enterovirus G (EV-G), Porcine Astrovirus 4 (PAstV4), Porcine Sapovirus (PSaV), Porcine Teschovirus (PTV), Posavirus (PoV), Rotavirus C (RVC), Porcine Parvovirus type 4 (PPV4), Porcine Adenovirus (PAV), Rotavirus B (RVB), Porcine Sapelovirus (PSV), Porcine Astrovirus 5 (PAstV5), Porcine Bocavirus (PBoV), Porcine Astrovirus 2 (PAstV2), Porcine Astrovirus 1 (PAstV1), Rotavirus H (RVH), Procine Torovirus (ToV), Porcine Parvovirus type 2 (PPV2) and Picobirnavirus (PbiV)

Notably, the sample pre-processing for nanopore analysis did not involve a nucleotide amplification step. Consequently, the semi-quantitative results from the nanopore sequencing, illustrated in Fig. 2, provided insights into the dynamics of various viruses in the fecal samples over time. Quantitative data on the total number of viral reads can be found in Additional File 2: Table S2, while the distribution of viral reads per age group of clinically healthy piglets is presented in Additional File 3: Figure S1. Of note, the total read counts per age group showed a similar trend as found with VIDISCA, with an increase at 5 weeks of age, followed by a downward trend up till 10 weeks of age. Picobirnavirus was detected in every age group, especially after weaning in high to very high amounts. A detailed analysis of the different viruses is presented below in the context of comparisons with qPCR analysis.

### Confirmatory qPCR-based analyses of selected viruses

For confirmatory analyses of the nanopore sequencing results, we selected Rotavirus A (RVA), Rotavirus C (RVC), Porcine Astrovirus types 1–5 (PAstV 1–5), Porcine Kobuvirus (PKoV), Porcine Sapelovirus (PSV), Enterovirus G (EV-G), and Porcine Sapovirus (PSaV). Individual sample analyses were conducted using this classical diagnostic method to gain insights into the dynamics of these viruses during the first ten weeks of life.

Importantly, qPCR data provided individual quantitative viral load measurements, which could be averaged across positive samples, and allowed calculation of the percentage of test-positive pigs. These parameters could not be derived from pooled sample analyses performed with nanopore sequencing. The qPCR data are presented with each farm as the independent statistical unit, since individual pig data demonstrated a clear dependency on farm and litter or group within each farm at every time point analyzed. For each farm, we calculated the average percentage of positive piglets and the average viral load in positive pigs based on individual sample analyses.

### Rotavirus nanopore and qPCRs

**Fig. 3:**
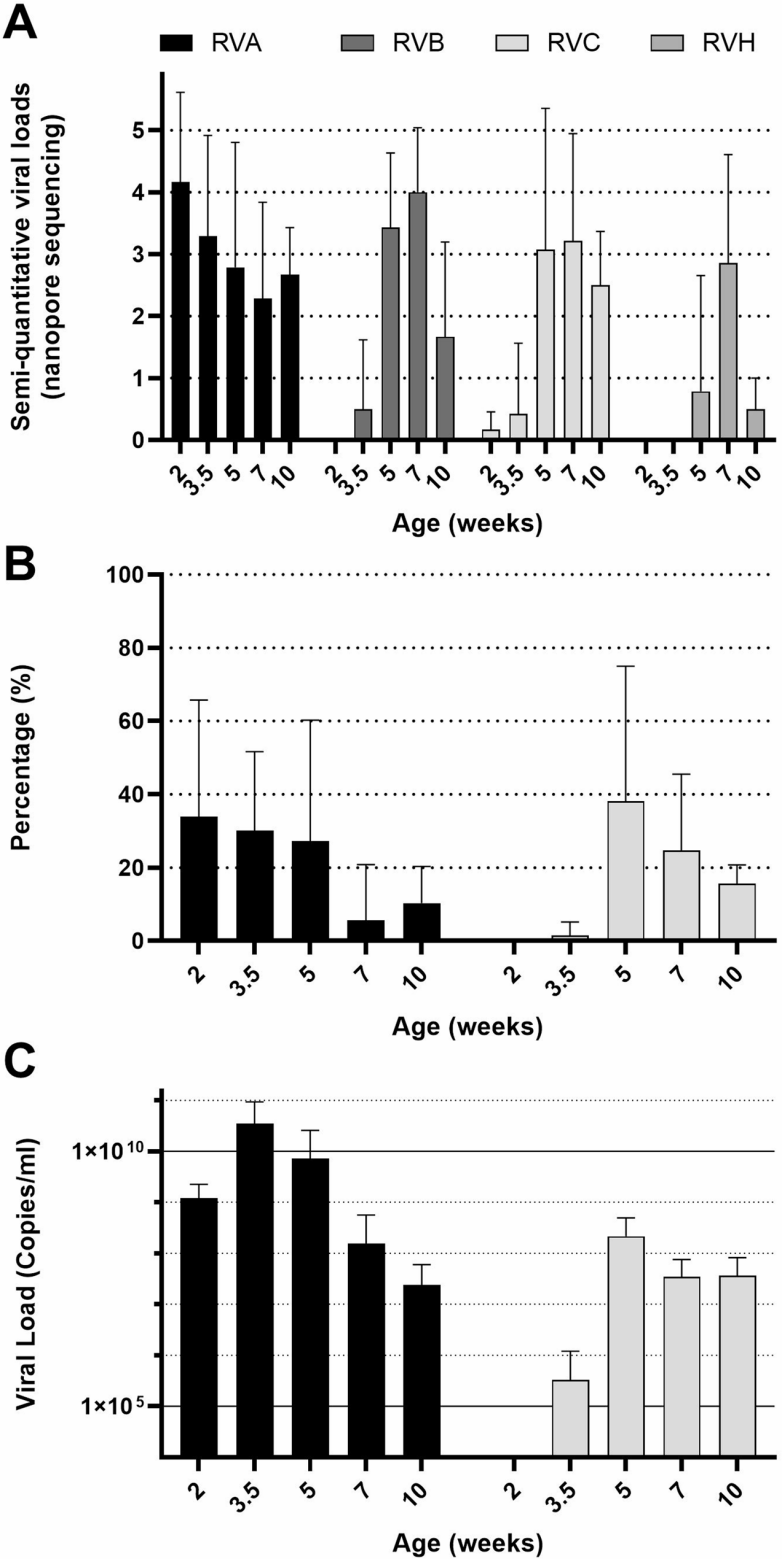
Rotavirus (RV) quantifications. **(A)** Average semi-quantitative scores of Nanopore sequencing across farms per age group, mean + SD **(B)** Average percentage positive piglets per age group across farms determined by qPCR for RVA and RVC, mean + SD. **(C)** Average viral load in the qPCR of the positive samples across farms for RVA and RVC, mean + SD

Rotavirus A (RVA), B (RVB), C (RVC), and H (RVH) were detected by nanopore sequencing across all farms, with the exception of RVH in farm 2 (see Figs. 2 and 3A, Additional file 2: Table S2,). Notably, the timing and pattern of first detection varied among RVA, RVC, RVB, and RVH; however, a remarkably consistent pattern emerged across farms. RVA was present in all age groups, while RVC became detectable from 3.5 weeks of age, peaking at 5 weeks, which is one week after weaning. RVB was identified one-week post-weaning, and RVH was detected from 7 weeks of age onward. The results of the individual qPCR analyses for RVA and RVC are shown in Figs. 3B (percentage positive) and 3 C (average quantitative load in positive samples). The highest viral load of RVA was observed just before weaning (3.5 weeks of age), whereas RVC exhibited its peak viral load immediately after weaning (5 weeks of age). Nanopore sequencing data analysis allowed for subtyping of RVA, revealing the presence of multiple genotypes in most farms (see Additional File 4: Figure S2 for a phylogenetic tree of RVA genotypes based on VP4 and Additional File 5: Figure S3 based on VP7). Up to three different genotypes were identified within a single farm, which showed sequential infections with overlap (see Additional file 6: Table S3).

### Astrovirus nanopore and qPCRs

**Fig. 4:**
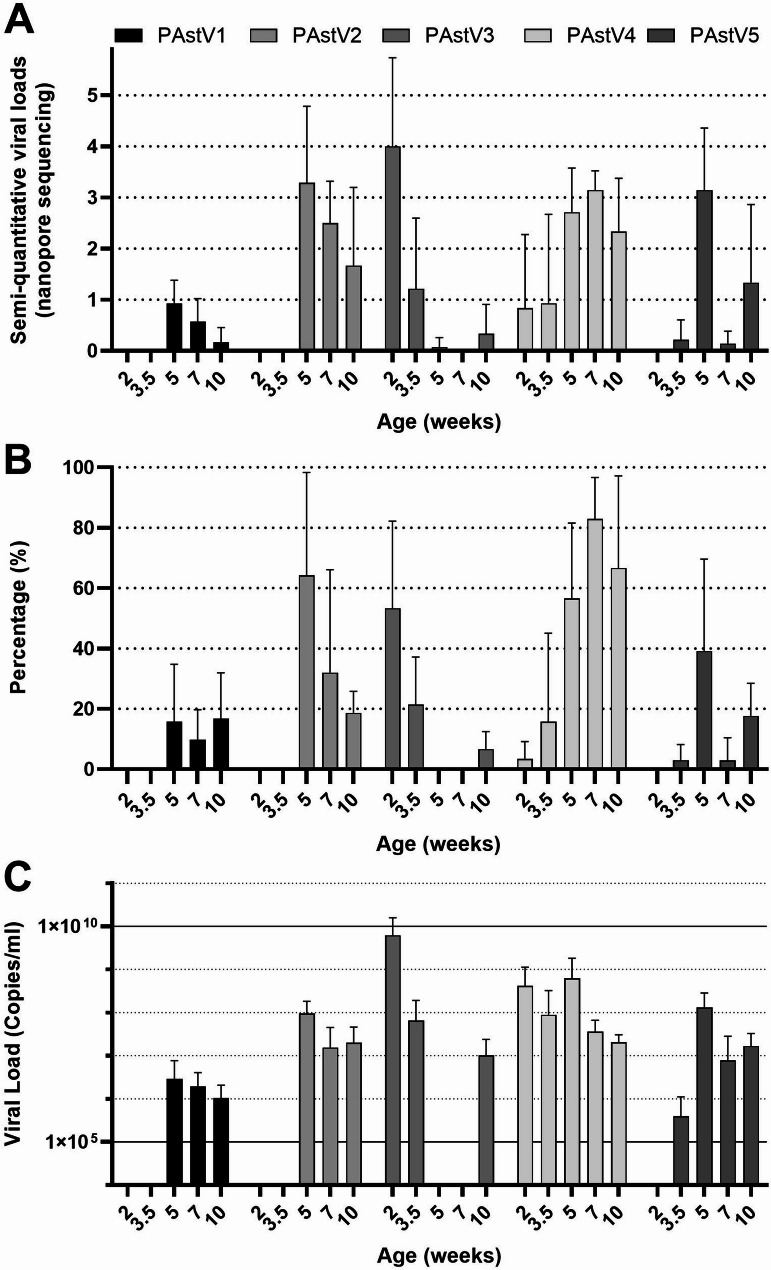
Porcine Astrovirus (PAstV) quantifications. **(A)** Average semi-quantitative scores of Nanopore sequencing across farms per age group, mean + SD. **(B)** Average percentage positive piglets per age group across farms determined by qPCR, mean + SD. **(C)** Average viral load in the qPCR of the positive samples across farms, mean + SD

Porcine Astrovirus was detected in all farms through nanopore sequencing (see Fig. 2 for individual farm scores; Fig. 4A provides average semi-quantitative viral loads per age group). Phylogenetic analysis of Porcine Astrovirus (PAstV) is presented in Additional File 7: Figure S4. Additionally, a set of five qPCR assays was employed to obtain quantitative data on PAstV1-5 infections. PAstV was detected by qPCR across all farms and age groups (see percentage positive scores in Fig. 4B and viral load in Fig. 4C), with distinct patterns observed for individual virus subtypes that, like the Rotavirus data, were remarkably consistent across farms. Types 3 and 4 were detected at 2 and 3.5 weeks of age, but PAstV3 was typically cleared by 5 weeks of age (one week after weaning). Concurrently, a shift towards types 1, 2, and 5 was noted. PAstV4 was present in every age group, with the percentage of positive pigs increasing over time. The peak viral load for Porcine Astrovirus type 3 was observed at 2 weeks of age, while the peak viral loads for types 1, 2, 4, and 5 occurred at 5 weeks of age (one week after weaning).

### Porcine Kobuvirus, Porcine Sapelovirus, Enterovirus G and Porcine Sapovirus, nanopore and qPCRs

**Fig. 5:**
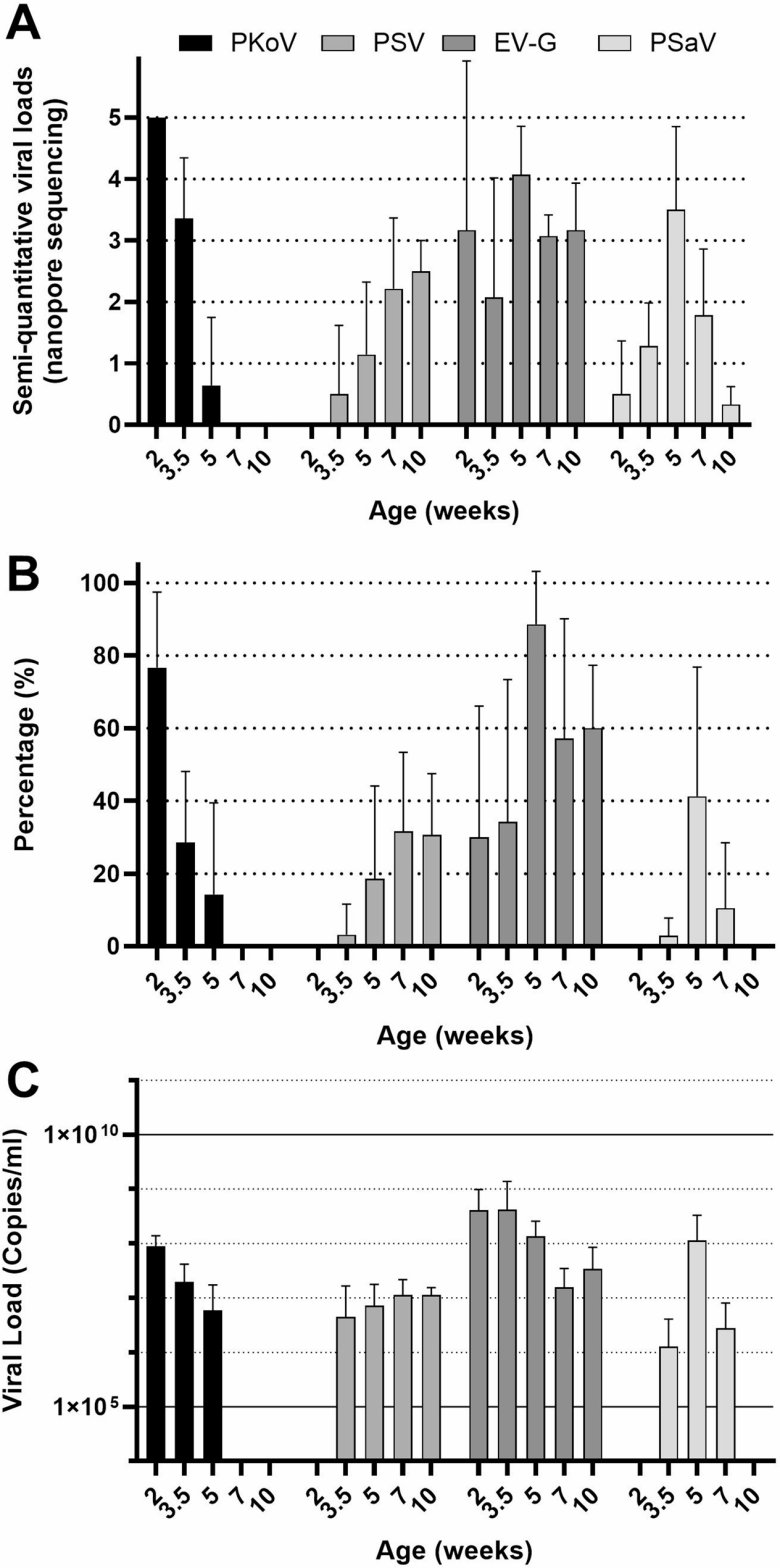
Porcine Kobuvirus (PKoV), Porcine Sapelovirus (PSV), Enterovirus G (EV-G) and Porcine Sapovirus (PSaV) quantifications. **(A)** Average semi-quantitative scores of Nanopore sequencing across farms per age group, mean + SD. **(B)** Average percentage positive piglets per age group across farms determined by qPCR, mean + SD. **(C)** Average viral load in the qPCR of the positive samples across farms, mean + SD

Porcine Kobuvirus was detected by nanopore sequencing in all farms, but it cleared quickly after weaning (see Figs. 2 and 5.A). Enterovirus G was identified at all farms across all age groups by nanopore sequencing. Individual qPCR-analyses showed the number of positive piglets increasing after weaning, peaking at 5 weeks of age (Fig. 5.B). In contrast, the viral load (determined by qPCR) before weaning tended to be higher than that observed after weaning (Fig. 5.C). Porcine Sapovirus was detected by qPCR in swabs starting from 3.5 weeks of age in a few piglets, with the highest number of positive piglets observed at 7 weeks of age. The viral load remained consistent across age groups. Porcine Sapelovirus was also detected in the first week after weaning. By 10 weeks of age, the viral load, based on the qPCR analyses, of Porcine Sapovirus (PSaV) decreased, while the load of Porcine Sapelovirus (PSV) remained more stable. This pattern was remarkably similar across farms. Based on the percentage of positive pigs at each time point, Porcine Kobuvirus (PKoV) and Enterovirus G (EV-G) were found to be more prevalent than PSV and PSaV.

## Discussion

This study aimed to elucidate the viral dynamics in enteric samples from clinically healthy young pigs during their first ten weeks of life. As the use of Next Generation Sequencing (NGS) technology in veterinary diagnostics continues to rise, we face the critical challenge of defining the normal virome composition in thriving animals. This understanding is essential, as the interpretation of NGS results relies on accurately distinguishing between a ‘normal’ and a ‘diseased’ state, a task that is not straightforward as the virome composition is critically influenced by the age of the animals.

To identify the landscape of viruses within the enteric virome of pigs, we used the VIDISCA NGS methodology, known for its high sensitivity [12]. Once we had established the viral landscape, we transitioned to a nanopore-based platform, which is nowadays more accessible to veterinarians due to its relatively low cost and rapid turnaround time—features that align well with the needs of end-users. To facilitate the use of this platform, pooled sample analysis of up to five samples per pool is likely to become standard practice. However, this approach may compromise the granularity of individual pig data. To enhance our scientific understanding of viral dynamics in well-performing herds, we conducted parallel qPCR analyses on individual samples and compared these results with those obtained from pooled nanopore sequencing.

We show in this study that the mere presence of a diverse set of potentially pathogenic viruses in the gastrointestinal tract may not immediately cause clinical signs, like diarrhea or wasting. For example, genotypes of RVA detected with significant viral loads in this study have been described to be at least potentially pathogenic [23]. Although a quantitative increase over time in the number of viral reads indicates active replication in the gastrointestinal tract, we have no insight in potentially associated tissue damage caused by infection of for example Rotaviruses in the pigs studied. Unfortunately, literature on virus presence in enteric samples with links to disease does not always present control data on enteric viruses present in healthy animals [2]. Other (longitudinal) studies compared the presence of multiple enteric viruses between healthy and diseased animals in rectal samples [5, 7] or in fecal samples [24]. The fact that potentially pathogenic viruses are present in diseased pigs as well as healthy pigs implies that other factors play a major role in the development of disease after infection.

We report a marked consistency in the patterns of Rotavirus infections across clinically ‘healthy’ farms. The nanopore sequencing analysis and qPCRs for Rotaviruses showed almost the same trends in viral infection over the first 10 weeks of life. However, important and previously unreported nuances can be made. RVA infections were primarily detected at the first three time points (2, 3.5 and 5 weeks of age) with one third to half of the pigs positive per farm. After weaning the percentage of positive piglets decreased, accompanied by declining RVA loads in the positive piglets. In experimental infections, shedding of RVA lasts for two weeks [25]. If we translate this to our farms, this may indicate that either RVA spread slowly between the piglets, or that multiple infections with different genotypes occurred sequentially. The detection of multiple VP4 and VP7 genotypes of RVA in the same farm - which has been described previously for piglets [26] but also for humans [6, 27, 28] - points towards multiple (partly overlapping) sequential infections during the first ten weeks of life.

RVB and RVC peaked in viral excretion and percentage of pigs infected at 5 and 7 weeks of age. RVH peaked at 7 weeks of age, somewhat later than RVB/RVC. To accurately access the viral excretion and the number of pigs positive for RVB and RVH, a qPCR analysis would have been of additional value. Unfortunately, it appears challenging to set up pan-RVB and pan-RVH qPCRs.

Besides Rotaviruses, also Porcine Astrovirus infections showed a remarkably consistent infection pattern across farms. PAstV3 peaked early at two weeks of age, with half of the piglets infected with relatively high excretion levels. The viral load and the percentage of positive pigs dropped quickly after three weeks of age. In contrast, the percentage of PAstV4 infected piglets was low in the pre-weaning groups, but rose quickly after weaning, with similar virus excretion levels compared to pAstV3. Relatively few pigs were infected with PAstV1 and PAstV5 in the post weaning period. PAstV2 infected about 50% of the pigs post-weaning. These dynamics of PAstV infections have not been reported previously. The potential impact of Astrovirus infection on intestinal health needs further study, as there is not enough conclusive information in the literature to prove that Astroviruses cause disease in pigs [2, 29]. Interestingly, the detection of the different types of Astroviruses associated with age, was in line with data from Denmark, in which also PAstV 3, 4 & 5 were detected in piglets in the first week of life [30]. In the USA, PAstV 1, 2, 4 & 5 were detected mostly in nursery pigs, however, the percentage of positive samples was somewhat lower compared to those in our study [19]. Also, the prevalence of PAstV3 was relatively low in the study from the USA [19].

Porcine Kobuvirus is a virus with a doubtful link with disease or pathogenicity [31]. The clinically healthy piglets in our study showed clear infections with PKoV at a relatively young age. The excretion of PKoV was almost exclusively observed before weaning and in a very high percentages of piglets per litter. A study of Theuns et al. in 2018 reported early shedding of PKoV as well [18]. Studies from other European countries, Vietnam and China [24, 30, 32, 33] found PKoV in all age groups, even in mature boars and sows. On the other hand, pigs with diarrhea from Spain presented a sharp PKoV decline with age, which we also observed [2].

EV-G was detected in about one-third of the piglets before weaning, but the percentage infected increased to 90% of the piglets one week post weaning. Average viral loads per pig were similar over time. Literature indicate that EV-G is globally distributed and mostly detected in the age group 0–3 months [32, 34–37], and although this age range is wide, it is in line with the results from our study.

Porcine Sapo- and Porcine Sapelovirus were detected around the time of weaning but in relatively few pigs. The dynamic picture for Porcine Sapovirus is in line with other epidemiological studies (reviewed in [38]). Compared to PKoV and EV-G, the percentage of positive piglets seems to be lower.

We observed that Picobirnaviruses constitute a major part of the viral genomes detected in young pigs, but it is questionable if these should be considered a true component of the enteric virome. Prominent levels of Picobirnavirus were evident from both NGS platforms, but no qPCR was performed to quantify the viral loads. Picobirnaviruses most likely infect prokaryotes (reviewed in [39]) and perhaps also the gastrointestinal tract of the pig itself. The increasing amount of Picobirnavirus found with the increasing age of the sampled piglets may be related to the colonization of the pig intestine by microbiota.

We realize that our study has limitations. First, we considered all piglets included in the study as clinically healthy piglets, but subclinical infections can of course not be excluded. Secondly, we performed a cross-sectional study on the farms, as the study design did not offer the possibility to collect samples from longitudinally followed piglets. We envision that future studies should follow individual healthy animals and confirm that multiple sequential enteric viral infections occur in individual animals pre- and post-weaning, combined with detailed performance and welfare data.

It remains difficult to explain the uniform chronological order of viral infections in clinically healthy animals, but this is at least partially related to characteristics of the viruses involved. Young piglets are exposed to viruses in their environment which includes secretions of the sow immediately after birth. It is well-reported that Rotaviruses cause neonatal diarrhea in the first week of life. We show that enteric viruses are commonly detected at two weeks of age despite the absence of diarrhea, but with evidence for infection (active viremia). The pattern is likely shaped by differences in infectivity, shedding, and stability in the environment of the enteric viruses. We cannot easily pinpoint these aspects. For some viruses, like Rotaviruses, it isn’t exactly clear whether the sow, as a carrier, or the environment is the source of infection [40]. Furthermore, the presence of different viruses in pens, compartments or stables can also play a role in shaping this pattern, especially when piglets are moved to another compartment after weaning.

The infection patterns, both before and after weaning, are also shaped by maternal immunity. Maternally derived immunity plays a role in timing of infections, progression of infections and consequently the severity of clinical signs, as described for example for RVA [25]. We observed a sharp increase in the total number of viral reads after weaning, which is an indication that the decrease of maternal antibodies takes of the brake for viral replication for at least some enteric viruses. It is known that the duration of the effect of maternal antibodies differs from virus to virus, and thus the pattern of appearance of virus infections is likely influenced by maternal antibodies. For Rotavirus A, lactogenic immunity explains part of the clinical protection against diarrhea during the farrowing period [23]. The importance of maternal antibodies before weaning is furthermore illustrated by studies in which colostrum deprived / caesarian derived piglets are challenged with PAstrV1 & PAstrV5 in order to investigate the role of maternally derived immunity [41, 42]. These studies show that clinical disease due to Astrovirus infections can be dampened by maternally derived antibodies. Also other studies suggest that lactogenic immunity protects young piglets from disease, despite the early infection by the virus [43, 44]. After weaning, lactogenic immunity gradually disappears, because maternally derived antibody levels decrease. It is not possible to deduct from our data if levels for example of Porcine Sapovirus initially rose after weaning due to declining maternal antibody levels, followed by specific immunity build-up, but this is likely. The same may be true for Porcine Sapelovirus where maternally derived antibodies may initially have provided protection [45, 46].

The earliest infections, pre-weaning, are clearly active, with production of large amounts of virus particles of PAstV3, PKV, RVA, and EV-G. Such infections with substantial virus production apparently do not cause major damage at this early age, which could be due to the presence of neutralizing antibodies or other lactogenic immunity provided by the sow in this period as discussed above. We hypothesize that these early infections may be of benefit to the animals. The actively replicating viruses will induce an innate immune response in the piglets and ultimately an adaptive immune response - with training and maturation of immune system - and by doing so the early infections could help to protect against severe disease in the post weaning period. At that time the pigs, encounter multiple viral infections. The fact that many viral infections have been cleared by the time the piglets reach the age of ten weeks may be illustrative for the build-up of protective immune responses. In this light, infection patterns and viremias observed may indicate an overall good herd health status, despite circulating pathogenic enteric viruses.

## Conclusions

In summary, we demonstrate that Next Generation Sequencing technology can serve as a powerful tool for the simultaneous detection of multiple viruses, opening new avenues for viral diagnostics in the veterinary field. We emphasize that enteric viral infections are present in clinically healthy pigs, and that the interpretation of virome composition is critically influenced by the age of the animals. This study provides a detailed qualitative and quantitative characterization of the enteric virome of clinically healthy piglets in the first ten weeks of life, during which a diverse set of viruses was detected but also cleared with age, with consistent patterns over multiple farms. These insights into the dynamics of the enteric virome are essential for correct interpretation of NGS-based diagnostics in the field.

## Electronic supplementary material

Below is the link to the electronic supplementary material.

Supplementary Material 1

Supplementary Material 2

Supplementary Material 3

Supplementary Material 4

Supplementary Material 5

Supplementary Material 6

Supplementary Material 7

## Data Availability

Data are property of MSD Animal Health; they can be made available however under reasonable request, but some restrictions might apply.

## Abbreviations

EV-G: Enterovirus G
NGS: Next generation sequencing
PAstV1: Porcine Astrovirus 1
PAstV2: Porcine Astrovirus 2
PAstV3: Porcine Astrovirus 3
PAstV4: Porcine Astrovirus 4
PAstV5: Porcine Astrovirus 5
PAV: Porcina Adenovirus
PbiV: Picobirnavirus
PBoV: Porcine Bocavirus
PKoV: Porcine Kobuvirus
PoV: Posavirus
PPV2: Porcine Parvovirus 2
PPV4: Porcine Parvovirus 4
PSaV: Porcine Sapovirus
PSV: Porcine Sapelovirus
PTV: Porcine Teschovirus
RVA: Rotavirus A
RVB: Rotavirus B
RVC: Rotavirus C
RVH: Rotavirus H
ToV: Porcine Torovirus
