# Supplementary material for: Characterization of the enteric virome of clinically healthy pigs around weaning on commercial farms in the Netherlands using next generation sequencing and qPCR
Source: Porcine Health Manag. 2025 Jul 24;11:41. doi: 10.1186/s40813-025-00446-5 (PMC12291374; doi:10.1186/s40813-025-00446-5)
Supplement: Supplementary file 1 — Supplementary Material 1 [file 40813_2025_446_MOESM1_ESM.docx]

Additional file 1: Table S1. Primer and probe sequences of the qPCRs used in this study including references.

|  | **Forward primer** | **Reverse primer** | **Probe** | **Ref** |
| --- | --- | --- | --- | --- |
| Astrovirus 1 | CTSYATGGGAAACTCCTKWGY | YTTTGGTCCKCCCCYCCAAA | 56-FAM/ATGGACGAG/ZEN/GAACATCCCTTCAAATGCT/3IABkFQ | Xiao et al. [19] |
| Astrovirus 2 |  |  | 5SUN/CCGGGCGCA/ZEN/CCTTGCCTAAAATC/3IABkFQ | Xiao et al. [19] |
| Astrovirus 3 |  |  | 56-FAM/TCCTTGGCC/ZEN/ATAACCTCCCTGATGACA/3IABkFQ | Xiao et al. [19] |
| Astrovirus 4 |  |  | 5SUN/AAATGTTTG/ZEN/GCTGAAACAGCGAGGCAC/3IABkFQ | Xiao et al. [19] |
| Astrovirus 5 |  |  | 56-ROXN/CTCGTGTTGGCTCTGATCTGCCAGTCTT/3IAbRQSp | Xiao et al. [19] |
| RVA 1 | GCTAGGGAYAAAATTGTTGAAGGTA | ATTGGCAAATTTCCTATTCCTCC | 6-Fam/ATGAATGGAAATGAYTTTCAAAC/BHQ1 | Marthaler et al. [22] |
| RVA 2 |  |  | 6-Fam/ATGAATGGAAATAATTTTCAAAC/BHQ1 | Marthaler et al. [22] |
| RVC | ATGTAGCATGATTCACGAATGGG | ACATTTCATCCTCCTGGGGATC | Texas Red/GCGTAGGGGCAAATGCGCATGA/BHQ1 | Marthaler et al. [22] |
| Kobuvirus | TCTCTGACCTCTGAAGTGCACT | TGAAGAAGCCATGTGTCTTGTC | 56-Fam/TGGTTGCGT/ZEN/GGCTGGGAATCCAC/3IABkFQ | Zhou et al. [23] |
| Enterovirus G | TAGATCGGGCTGATGGGT | TGGTTAGGATTAGCCGCATT | 56-Fam/AGTGACAGGGTGTGAAGAGCCTATT/36-TAMSp | This study |
| Sapovirus | AGAAGTGTTCGTGATGGAGGCG | CTRACCACACTGGGGATGGT | 56-Fam/CAACCCGTYCGGTKGCGAGCAAYCCAGA/36-TAMSp | This study |
| Sapelovirus | GTGGTAGGGATTTAGTTTTGATTGA | TGCCAATGGTACCAGCACAT | 56-Fam/TATGGTGATGATTTATTGGTTGCCTATCCCTA/36-TAMSp | This study |
