# Supplementary material for: Characterization of the enteric virome of clinically healthy pigs around weaning on commercial farms in the Netherlands using next generation sequencing and qPCR
Source: Porcine Health Manag. 2025 Jul 24;11:41. doi: 10.1186/s40813-025-00446-5 (PMC12291374; doi:10.1186/s40813-025-00446-5)
Supplement: Supplementary file 2 — Supplementary Material 2 [file 40813_2025_446_MOESM2_ESM.docx]

Additional file 2: Table S2. Overview of the number of viral reads per age group of clinically healthy piglets from nanopore sequencing.

| **Virus** | **2 weeks of age** | **3.5 weeks of age** | **5 weeks of age** | **7 weeks of age** | **10 weeks of age** |
| --- | --- | --- | --- | --- | --- |
| RVA | 580584 | 597965 | 424709 | 108118 | 2228 |
| RVB |  | 753 | 91156 | 138032 | 323 |
| RVC | 27 | 726 | 315405 | 76466 | 28263 |
| RVH |  |  | 34184 | 168193 | 51 |
| PAstV1 |  |  | 561 | 181 | 23 |
| PAstV2 |  |  | 16519 | 10280 | 337 |
| PAstV3 | 101759 | 6709 | 23 |  | 25 |
| PAstV4 | 3851 | 2327 | 11224 | 7754 | 974 |
| PAstV5 |  | 201 | 19105 | 82 | 186 |
| PKoV | 59462 | 38934 | 2178 |  |  |
| PSV |  | 341 | 1859 | 7217 | 872 |
| EV-G | 24463 | 46798 | 32648 | 16680 | 1756 |
| PSaV | 364 | 1252 | 55141 | 2783 | 25 |
| PboV |  | 15 | 520 | 1342 | 209 |
| PbiV | 311 | 74796 | 62717 | 92966 | 26656 |
| ToV |  |  | 2325 | 1389 | 138 |
| PTV | 760 | 588 | 1105 | 308 | 173 |
| PoV | 4 | 3495 | 2639 | 403 | 774 |
| PPV2 |  |  |  | 10 |  |
| PPV4 | 1 |  |  | 1 | 2 |
| PAV |  | 4470 | 47 | 10 | 38 |
