# Supplementary figures and images for: Characterization of the enteric virome of clinically healthy pigs around weaning on commercial farms in the Netherlands using next generation sequencing and qPCR
Source: Porcine Health Manag. 2025 Jul 24;11:41. doi: 10.1186/s40813-025-00446-5 (PMC12291374; doi:10.1186/s40813-025-00446-5)

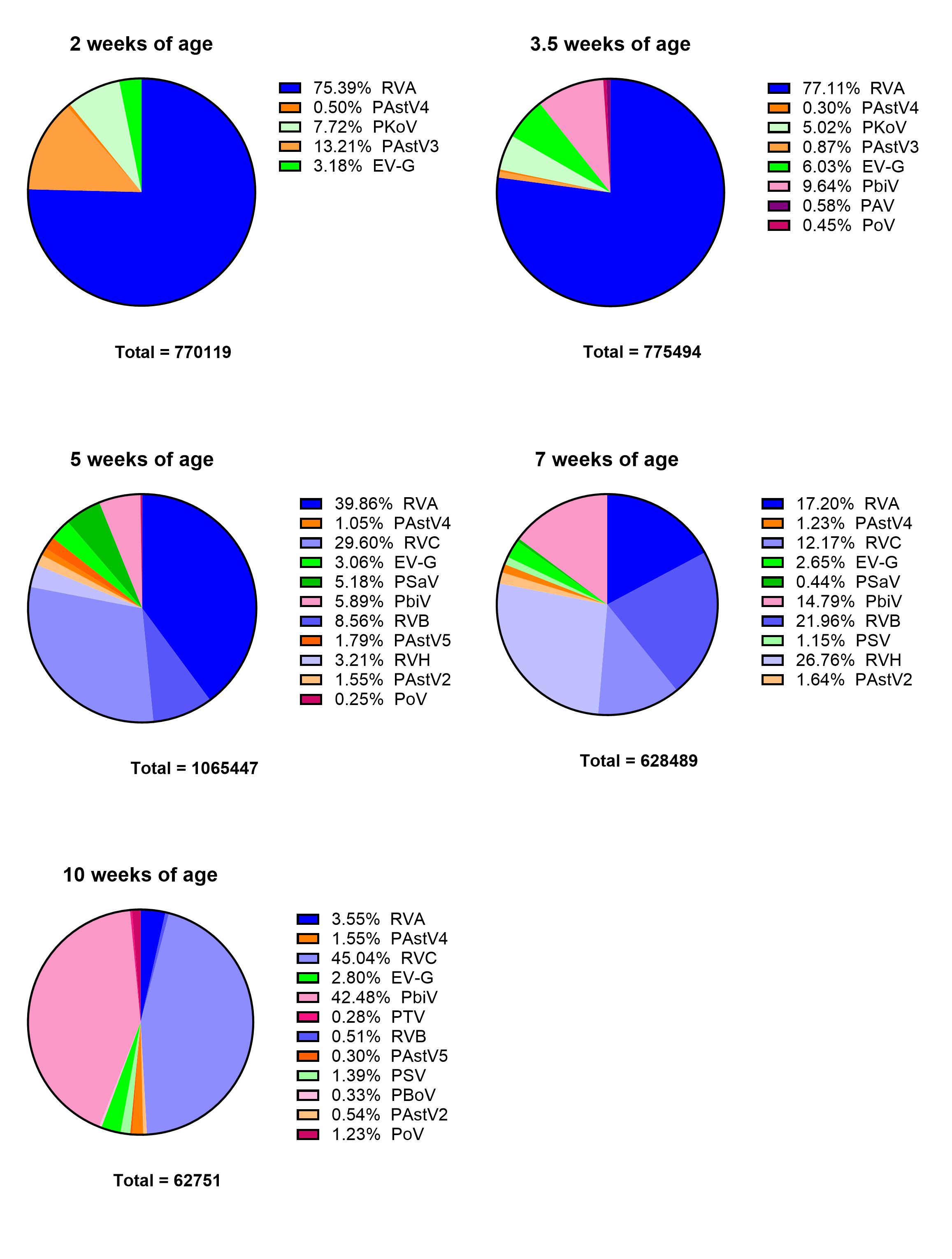

Supplement: Supplementary file 3 — Supplementary Material 3 [file 40813_2025_446_MOESM3_ESM.jpg]

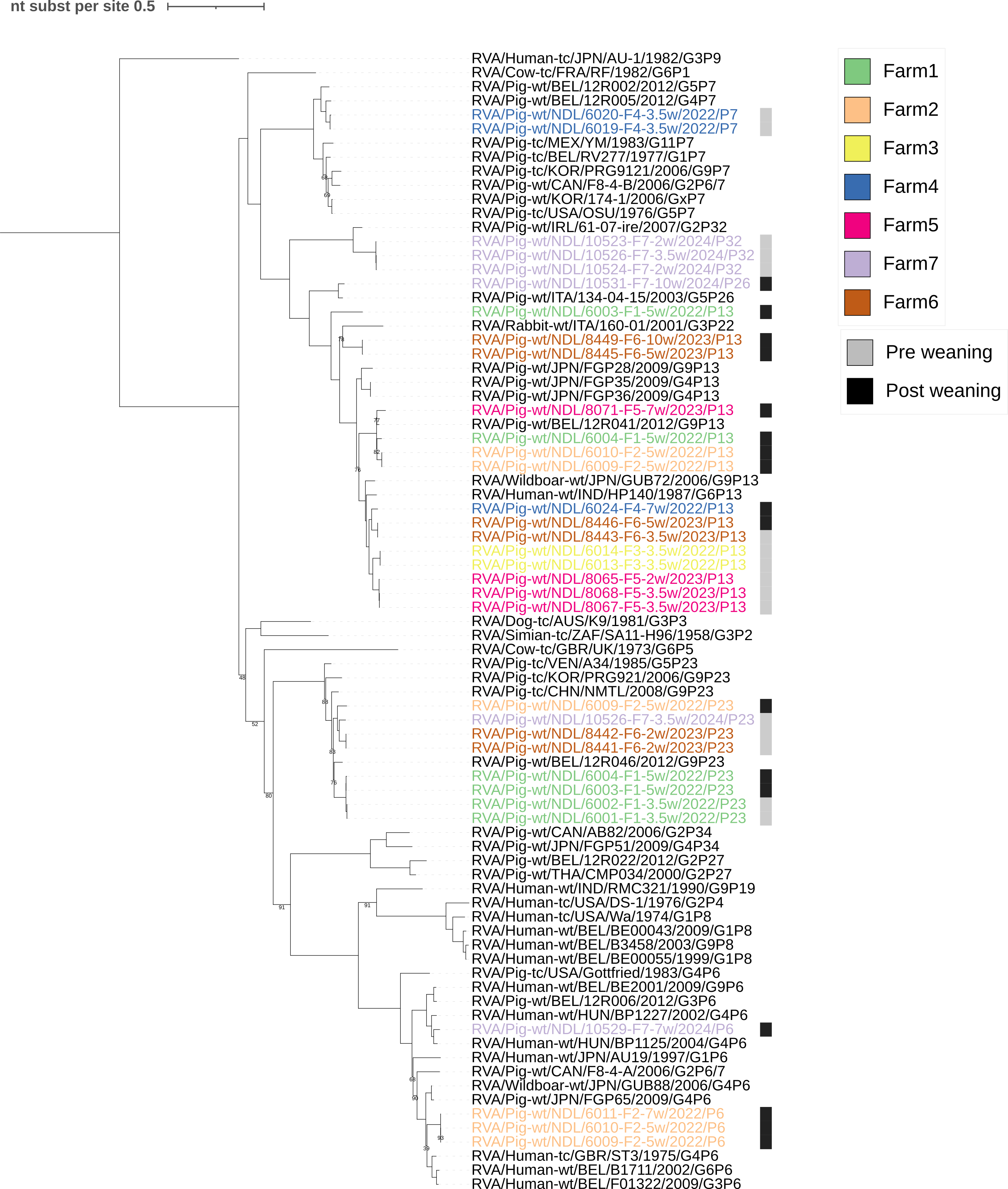

Supplement: Supplementary file 4 — Supplementary Material 4 [file 40813_2025_446_MOESM4_ESM.png]

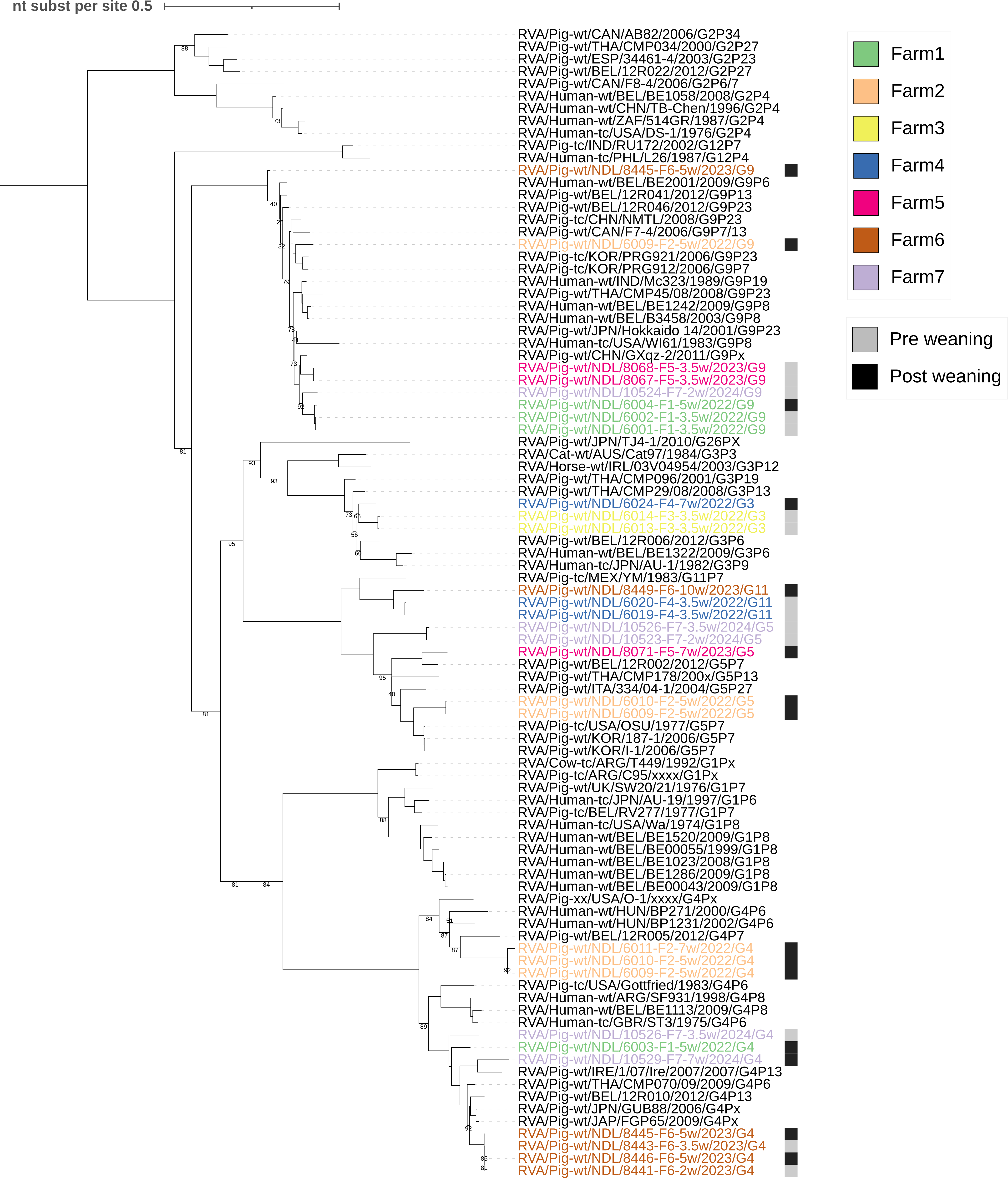

Supplement: Supplementary file 5 — Supplementary Material 5 [file 40813_2025_446_MOESM5_ESM.png]

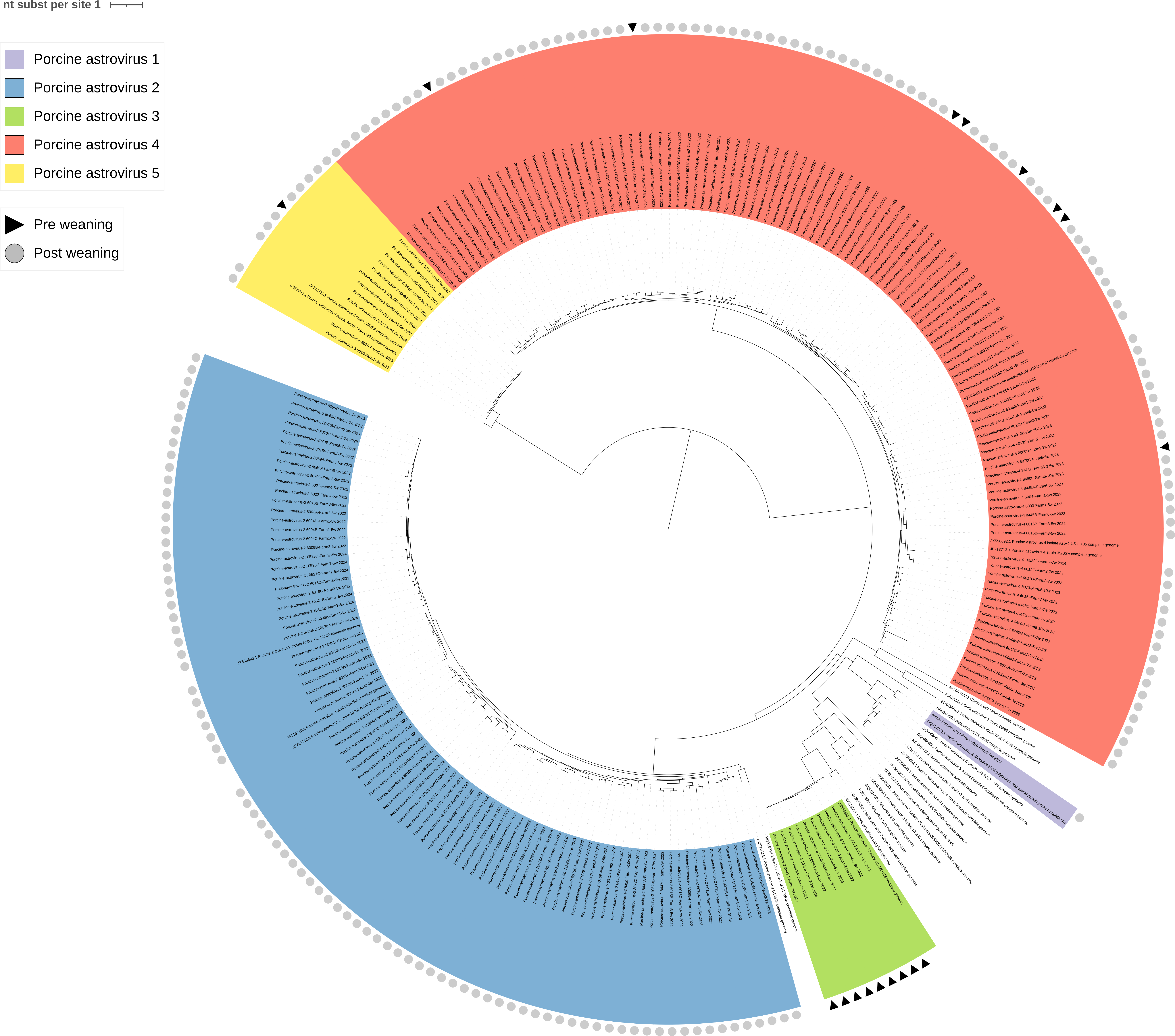

Supplement: Supplementary file 7 — Supplementary Material 7 [file 40813_2025_446_MOESM7_ESM.png]
