# Supplementary material for: Characterization of the enteric virome of clinically healthy pigs around weaning on commercial farms in the Netherlands using next generation sequencing and qPCR
Source: Porcine Health Manag. 2025 Jul 24;11:41. doi: 10.1186/s40813-025-00446-5 (PMC12291374; doi:10.1186/s40813-025-00446-5)
Supplement: Supplementary file 6 — Supplementary Material 6 [file 40813_2025_446_MOESM6_ESM.docx]

Additional file 6: Table S3. Detected genotypes of RVA in piglets per age group per farm.

|  |  | **2 weeks of age** | **3.5 weeks of age** | **5 weeks of age** | **7 weeks of age** | **10 weeks of age** |
| --- | --- | --- | --- | --- | --- | --- |
| Farm 1 | VP7 |  | G9 | G4/G9 | not detected |  |
|  | VP4 |  | P23 | P23/P13 | not detected |  |
| Farm 2 | VP7 |  | not detected | G4/G9/G5 | G4 |  |
|  | VP4 |  | not detected | P23/P6/P13 | P6 |  |
| Farm 3 | VP7 |  | G3 | not detected | not detected |  |
|  | VP4 |  | P13 | not detected | not detected |  |
| Farm 4 | VP7 |  | G11 | not detected | G3 |  |
|  | VP4 |  | P7 | not detected | P13 |  |
| Farm 5 | VP7 | not detected | G9 | not detected | G5 | not detected |
|  | VP4 | P13 | P13 | not detected | P13 | not detected |
| Farm 6 | VP7 | G4 | G4 | G4/G9 | not detected | G11 |
|  | VP4 | P23 | P13 | P13 | not detected | P13 |
| Farm 7 | VP7 | G5/G9 | G4/G5 | not detected | G4 | not detected |
|  | VP4 | P32 | P23/P32 | not detected | P6 | P26 |
